# Artificial Intelligence, Responsibility Attribution, and a Relational Justification of Explainability

**DOI:** 10.1007/s11948-019-00146-8

**Published:** 2019-10-24

**Authors:** Mark Coeckelbergh

**Affiliations:** Department of Philosophy, University of Vienna (Universität Wien), Universitätsstrasse 7 (NIG), 1180 Vienna, Austria

**Keywords:** Artificial intelligence (AI), Responsibility, Responsibility attribution, Responsibility conditions, Answerability, Moral agency, Moral patiency, Problem of many hands, Transparency, Explainability

## Abstract

This paper discusses the problem of responsibility attribution raised by the use of artificial intelligence (AI) technologies. It is assumed that only humans can be responsible agents; yet this alone already raises many issues, which are discussed starting from two Aristotelian conditions for responsibility. Next to the well-known problem of many hands, the issue of “many things” is identified and the temporal dimension is emphasized when it comes to the control condition. Special attention is given to the epistemic condition, which draws attention to the issues of transparency and explainability. In contrast to standard discussions, however, it is then argued that this knowledge problem regarding agents of responsibility is linked to the other side of the responsibility relation: the addressees or “patients” of responsibility, who may demand reasons for actions and decisions made by using AI. Inspired by a relational approach, responsibility as answerability thus offers an important additional, if not primary, justification for explainability based, not on agency, but on patiency.

## Introduction

In response to recent progress and successes in artificial intelligence (AI), especially machine learning applications, ethics of AI has become a popular topic in academic and public discussions about the future of technology. Academic contributions range from speculations about superintelligence (e.g. Bostrom 2014) to discussions about specific, arguably more near-future technologies and phenomena such as self-driving cars (e.g. Helveke and Nida-Rümelin 2015; Nyholm and Smids 2016). There are public discussions about, for example, the future of work and about privacy. Or to give another example from the transportation sector: consider the recent discussion about automation in airplanes following the recent crashes of Boeing 737 MAX aircraft, which are probably related to malfunctioning software, with pilots trying to override the software.^1^

One of the topics that needs more discussion is responsibility. Given that AI enables society to automate more tasks and automate to a larger extent than before, who or what is responsible for the benefits and harms of using this technology? And, if this problem should be tackled pro-actively in the domains of technology and policy, what does the development of “responsible AI” mean? This is not only a philosophically interesting, but also a very practical, problem that urgently needs more conceptual work. It is also relevant to ethics of (other) automation technologies such as robotics, although there are some issues that are specific to some AI (e.g. the so-called “black box” problem which will be mentioned below).

This paper focuses on the question of responsibility attribution for artificial intelligence technologies used in the automation of actions and decisions usually made by humans. But the attribution discussion also points to a problem that is not only about the *agents* of responsibility but also about the *patients* in the responsibility relation.

Here is a summary of the paper. The problem of responsibility attribution is initially approached by distinguishing between two Aristotelian conditions of responsibility, one is about control and a need to identify the responsible agent, and another which examines the agent’s knowledge. First, it is assumed that, even if AI technologies gain more agency, humans remain responsible since only the latter can be responsible: artificial intelligence technologies can have agency but do not meet traditional criteria for moral agency and moral responsibility. Nevertheless, there are many challenges with regard to responsibility attribution and distribution, not only due to the problem of “many hands” but also due to what I call “*many things*”. It is also important to take into account the *temporal* dimension when it comes to causes. Special attention is then given to problems about knowledge related to some machine learning applications which have consequences for responsibility. Usually such problems are discussed in terms of transparency and explainability. However, in contrast to most discussions in the literature and in public settings, it is added that the problem of the addressee should not be neglected when it comes to explainability: those *to whom* moral agents are responsible. Seen from a more relational perspective, there are not only moral agents but also *moral patients* in the responsibility relation. It is argued that the demand for explainability is justified not only via the knowledge condition (know what you are doing as an agent of responsibility) but should also be based on the moral requirement to provide reasons for a decision or action to those to whom you are answerable, to the responsibility patients. Finally, other senses of the term “relational” responsibility are also explored: collective responsibility and the social aspect of giving explanations.

Note that I am aware that *starting* from an Aristotelian approach, like all standard approaches to responsibility, has its limitations and constraints. For example, it assumes that technologies are mere tools and instruments, starts from the premise that only humans can be responsible agents, tends to focus on individual responsibility, and focuses on the agent of responsibility rather than the patient of responsibility. Some of these assumptions, like instrumentality and the view that only humans can be responsible agents, I will mention but do not discuss in detail for the sake of limiting the scope of my paper; I have addressed these topics elsewhere. Other assumptions I *do* challenge in this paper: the relational emphasis enables a more social and patient-oriented approach than standard approaches.

I acknowledge that a discussion of legal concepts and problems is very helpful to tackle practical and philosophical problems concerning responsibility for AI. For example, Turner (2018) provides a helpful overview of responsibility problems in relation to a number of legal instruments in private and criminal law. This is also very interesting and relevant but falls beyond the scope of this paper.

In the course of the paper I will mainly give examples from the domain of transportation, in particular self-driving cars and airplanes, but what is said here is applicable beyond those domains to all uses of AI and often even to all kinds of automation technologies and AI applications. My main aim is not to shed light on particular cases but to contribute to a general conceptual framework for thinking about responsibility for AI and other advanced automation technologies, with a focus on AI as machine learning.

## The Problem of Responsibility Attribution: Who or What is the Agent of Responsibility?

It is important to be able to ascribe responsibility when something goes wrong with AI. For example, in the case of a self-driving car or an airplane using AI, it should be asked: if the automation system of the car or the airplane autopilot cause an accident, who is responsible? Next to this backward-looking responsibility one can also ask, in a forward-looking way: how can the technology and its social environment be shaped in a way that ensures that AI is used responsibly? For example, how can people develop AI-based cars and airplanes in such a way that helps to *prevent* accidents? Although my discussion will mainly focus on responsibility attribution related to the use of AI, it also has consequences for the responsible development of AI.

When humans are acting and making decisions, agency is normally connected with responsibility. You have an effect on the world and on others, and therefore you are responsible for what you do and for what you decide. Yet it is not always clear to whom to ascribe the responsibility. It may not be clear who precisely caused the relevant consequences (e.g. the harm but it could also be the benefit) and even if it is clear who did it, maybe the person was not acting voluntarily or did not know what she was doing. So how, to whom, and when can individuals and society meaningfully ascribe responsibility? And what does that mean for responsibility attribution in the case of AI?

A helpful way to approach the question concerning responsibility attribution is to start with the conditions of responsibility. Since Aristotle there are at least two traditional conditions for attributing responsibility for an action, the so-called ‘control’ condition and the ‘epistemic’ condition (Fischer and Ravizza 1998; Rudy-Hiller 2018). You are responsible if (1) you do it (or have done it)—if you are the agent of the action, if you have caused the action, if you have a sufficient degree of control over the action—*and* (2) if you know what you are doing, if you are aware what you are doing (or knew what you were doing). Aristotle argued in the *Nicomachean Ethics* (1984, 1109b30–1111b5) that the action must have its origin in the agent and that one must not be ignorant of what one is doing.

How do these conditions (agency/control and knowledge) fare in the case of the use of artificial intelligence technologies?

Let us start with the first condition, the so-called ‘control’ condition. In the modern literature, there is especially a lot of emphasis on individual control: an agent is responsible for an action or decision if and only if she has control over her action (e.g. Fischer and Ravizza 1998; McKenna 2008). Intent and freedom are usually linked to the control condition. Aristotle already stressed that an action needs to be voluntary, not forced. For example, if a human driver causes an accident, then it will be questioned if that driver actually caused the accident, if the particular action was voluntary and—now for the epistemic conditions—if she could have made a different choice (if there was an alternative), and if the driver knew what she was doing (e.g. was she texting, voluntarily, but ignorant of the impact of that action?).

Now all this assumes that the agent of responsibility is human. What about an agent based on AI? In response to the fact that artificially intelligent technologies gain more agency, it might be tempting to explore whether the technologies themselves can be responsible agents. In ethics of computing and robot ethics there have been extensive discussions about this topic and the related issue of moral agency (e.g. Sullins 2006; Johnson 2006; Sparrow 2007; Stahl 2006; Moor 2006; Wallach and Allen 2009; Floridi and Sanders 2004; Coeckelbergh 2010). Similar arguments could be offered for or against the idea that AI technologies can be responsible agents. Moreover, in response to the control problems, one may also take a hybrid approach and conceptualize responsibility as distributed across a network of humans and machines (Gunkel 2018a). For example, influenced by Latour, Hanson (2009) has argued for attributing responsibility to cyborgs and actor networks.

However interesting these proposed solutions are, I will not follow these routes here. While acknowledging that (1) technology shapes human action in a way that goes beyond a merely instrumental role, that (2) the actions of AI driven technology can be morally relevant (without going as far as suggesting that morality itself is technological, as Verbeek 2006 did), and that (3) advanced AI technologies may give the appearance of being responsible agents (Coeckelbergh 2009), the remainder of this paper will assume that AI technologies do not meet traditional criteria for full moral agency (and hence *pre*conditions for responsibility) such as freedom and consciousness, and that therefore they also cannot be (held) responsible. With regard to the two Aristotelian conditions, it is thus assumed that it *does not make sense* to demand that the AI agent act voluntarily or without ignorance, since an AI agent lacks the preconditions for this: an AI cannot really act “freely” (as in: “having free will”) or “know” (as in “being aware of”) what it is doing. Whether this is so for metaphysical reasons or simply a matter of social convention which deserves further critical questioning is not a concern here; I assume it in order to clear the ground for further arguments in this paper.

If this assumption holds, then our only option is to make humans responsible for what the AI technology does. Even if (some) AIs can act or decide (i.e. have agency), they lack the capacities for moral agency, and so the responsibility for their actions or decisions—actions and decisions delegated to them by humans—remains and should remain with the human agents who develop and use the technology. And this is in fact what is done in current legal systems when it comes to non-human things in general. Although some organizations can have legal personhood, their “responsibility” remains traceable to the responsibility of human beings. Technological agents are thus assumed to be a-responsible: they cannot be responsible (and therefore also not irresponsible). Humans, however, *can* be responsible and should be held responsible for what they do and decide when developing or using AI.

However, assuming this position does not solve the problem of responsibility attribution: even if only humans can be responsible agents, it is still an open question if humans *can* always take responsibility for the use of AI and *which* humans are responsible for the use of AI. Let me give an overview of some responsibility attribution issues related to the control/agency condition.

First, what if humans do not have sufficient control over the use of AI? This is what Matthias (2004) has called the ‘responsibility gap’ with regard to autonomously learning and acting machines: how can humans be held responsible when they have no or insufficient control? For example, if we hand over control of financial transactions to machines, as in high frequency trading, then how can humans be responsible for these transactions? And if autonomous driving means that humans no longer have the time to intervene, who is responsible when an accident happens?

Often this is indeed a problem of time. Consider a military situation in which an automated defense system is the only one that is fast enough to react to an attack: when there is a short-warning attack (or indeed no warning), humans cannot respond in time and now already autonomous modes are used in these situations (Horowitz and Scharre 2015). Consider for instance an automated anti-missile system: in case of an attack, human decision-making may be too slow, so automation is used. Cyber-attacks give rise to similar problems; software is then used that can intervene quickly and autonomously to protect the systems before harm is done.

How can humans take responsibility for such actions and decisions in such cases, given that the situation and the way this automation is set up means they cannot make an “end-decision”? It is not clear how one can deal with this at all, and therefore whether one should use these kinds of systems at all: whether society should create situations and develop technologies that, when it comes to the actual operations, no longer have room for human agency. What if there is no human “in the loop”, and what if it doesn’t even make sense anymore to say that there should be humans “*on* the loop” (supervising) since they cannot intervene anyway? It seems that the use of automation technology is moving in that direction in many domains. For example, in large commercial aircraft autopilot is used almost all the time, but when something goes wrong, the pilots cannot always intervene in time (consider the Boeing 737 max crashes). Are such automation systems and AIs ethically acceptable, or should there be limits to automation?

Second, one of the problems with technological action is that there are usually many people causally involved in the action, which renders it difficult if not impossible to (a) find *the* responsible individual, if there is only one individual responsible, or (b) hold any one *individual* responsible: there may be more people responsible since there are so many agents involved. This issue with responsibility attribution and distribution is sometimes referred to as “the problem of many hands” (e.g. van de Poel et al. 2012). This problem is also raised by AI applications. Consider for example the March 2018 self-driving car accident in Arizona: an Uber car caused the death of a pedestrian. Who is responsible? It could be the developers of the software, the car company that produced the car, Uber that employed the car, the car user, the pedestrian, and even the regulator (here the State of Arizona). And within the category “software development”—like in the airplane crashes, the software seems to have failed, having identified the pedestrian as ‘a false positive’^2^—there may be a lot of people involved.

In response, some authors have proposed the concept of distributed responsibility. Taddeo and Floridi (2018) write:

‘The effects of decisions or actions based on AI are often the result of countless interactions among many actors, including designers, developers, users, software, and hardware…. With distributed agency comes distributed responsibility’ (Taddeo and Floridi 2018, 751).

This seems a good conceptual solution to the problem of many hands. However, acknowledging the distributed character of responsibility in the case of AI does not solve the practical problem of *how* to distribute the responsibility, given that one may not know (the extent of) all the contributions and interactions, and given a number of other challenges. First, there are usually many parties involved, but some may be more responsible than others. For example, in the case of Microsoft’s 2016 Twitter chatbot Tay (a controversial chatbot which, after interaction with users, started to produce racist and misogynistic comments and had to be shut down), there were many parties involved: developers, designers, but also the company and those interacting with the chatbot. But in their analysis of the case, Suárez-Gonzalo et al. (2019) point mainly to the designers, developers, and those who decided to insert the bot, rather than the Twitter users who interacted with the bot. While it is not entirely clear to me why the latter would be a lot less responsible, it is clear that distributed responsibility does not imply that responsibility is and should always be distributed equally. Second, responsibility attribution may be difficult because one or more parties may have an interest in misrepresenting their contribution and in trying to evade responsibility. Suárez-Gonzalo et al. (2019) rightly emphasize the social character of what happened and identify some of the interests at play and the role of the media. In this case, they argue, the media discourse served the interests of Microsoft (Suárez-Gonzalo et al. 2019, 8). Similarly, in the transportation sector there are also many parties involved and different parties may have different interests when it comes to presenting their part in, for instance, an accident with an airplane or a self-driving car. Again, this renders responsibility attribution difficult in practice. Third, it is also important to know who did what *when*. Accident investigations such as in the airline industry try to reconstruct what happened at what time and who did what. This brings us to the next issue.

Third, the problem of responsibility attribution and distribution has a temporal dimension: who does what at what time (and where)? This can refer to the use of the technology and specific operations, for example who does what at what time in the cockpit of an airplane (e.g. to deal with an autopilot software problem), but it can also extend to the development of the technology. In the case of technology use and development, there is often a long causal chain of human agency. In the case of AI this is especially so since complex software often has a long history with many developers involved at various stages for various parts of the software. This can happen as software moves across organizations (e.g. a company) or even within the same organization. And in the case of machine learning AI there is also a process and history of the production, selection, and processing of data and datasets—again, with not just *one* human agent involved and happening at various times and places. There are people who give their data (voluntarily and knowingly or not), people who collect and process data, people who sell data, people who analyze data, etc. AI software may also be developed in and for one context of application, but later used in an entirely different context of applications. For example, in principle “medical” face recognition software can also be used for surveillance purposes and become “police” AI. Furthermore, there is not only use and development of technology, but also maintenance. If maintenance is not done responsibly, a technological system with AI included (say, an airplane) may also fail. This causal history with its many hands should be examined in order to attribute responsibility. But in the case of AI such an investigation may be very challenging. If there is no record and if this cannot be traced, there is a responsibility attribution problem. This is why traceability (Dignum et al. 2018; Mittelstadt et al. 2016) is an important way to operationalize responsibility and explainability (European Commission AI HLEG 2019).

But there are not only many (human) hands; there are also what one could call “many things”: many different technologies. In AI process and history, various software is involved but also more literally various things, material technological artefacts: things that are relevant since they causally contribute to the technological action, and that may have some degree of agency. There are many interconnected elements. For example, a malfunctioning sensor interacting with the software of an airplane may causally contribute to its crash; it is then important to find out how the technological system as a whole is structured and who is responsible for the development, use, and maintenance of its parts (for example a sensor) and the interaction between the parts. For example, in the case of the Lion Air flight 610 crash it has been suggested that a sensor had malfunctioned.^3^ And again the temporal dimension is important here: software often has long causal histories, but also the production and use of the hardware generally involves many artefacts (and hence producers, developers etc.). Consider an airplane or a contemporary car, which has many components and involves many interactions between these components. For questions regarding responsibility, forward-looking and backward-looking, it is important to clarify all these structural and temporal relations and interactions: not only the social interactions and roles of humans but also their interactions with things and the relations and interactions *between* things. In this sense, responsibility for technology is not only a matter of faces but also of interfaces.

This also means that it is not always clear if “the AI” was the cause of the problem, since it may be another component of the technological system, in itself not AI, that caused the problem in its interaction with AI—consider again the example of the malfunctioning sensor. It might also be a problem of (software or hardware) connections and interfaces between parts. And it is not clear where AI ends and the other technology begins. It is not even clear what “AI” is. Often the term “AI” is used for smart algorithms in general. There are ongoing discussions about the definition of AI in science, philosophy, and policy making. But regardless of these discussions about what AI is, for responsibility attribution it is important to clarify precisely what technical components are involved in a technological system, how they interact and interface, and how they contribute(d) to a problem and relate to human actors.

Finally, it is questionable how “voluntary” and “free” the use of AI is when considering the end-user, given that this user might not understand AI or might not even know *that* she uses AI (or indeed is used by AI). This leads us to questions concerning knowledge.

## Knowledge Problems: Transparency and Explainability

The second Aristotelian condition is the ‘epistemic’ condition (Fischer and Ravizza 1998, 13): one must know what one is doing, or negatively formulated by Aristotle: one must not be ignorant. More precisely, Aristotle distinguishes between a number of ways in which one can be ignorant:

‘A man may be ignorant, then, of who he is, what he is doing, what or whom he is acting on, and sometimes also what (e.g. the instrument) he is doing it with, and to what end (e.g. for safety), and how he is doing it (e.g. whether gently or violently). (Aristotle 1111a3-5)

Rudy-Hiller (2018) translates the requirement of non-ignorance in terms of awareness, and distinguishes between a number of kinds of awareness that have been distinguished by contemporary (analytic) philosophers: awareness of the action, awareness of the moral significance of the action, awareness of the consequences of the action, and (according to some) awareness of alternatives. But, interestingly, Aristotle also includes an element that concerns knowledge about the *instrument*: ‘what … he is doing it with’. This element is often ignored by philosophers but should be of concern to philosophers of technology: knowing the technology you are using is also important for responsibility.

Now these philosophical analyses of ignorance can guide discussions about knowledge problems with AI. In what sense do people involved in the use and development of AI know or not know what they are doing?

Usually programmers and users know what they want to do with the AI. More precisely, they know what they want the AI to do for *them*. They know the goal, the *intended* consequences; Aristotle would say the *end*. However, users of AI are not necessarily aware of the *non*-*intended* consequences and *moral significance* of what they do. For example, they may not know that there is a bias in the dataset they are using or even in their algorithm. They may not know their own biases and that these biases flow into the design of the algorithm. They may not even be aware that what they do is morally significant at all. (Note that it is also possible that the human expert is biased and the algorithm does better, as Sunstein 2018 has argued; algorithms can also help to detect discrimination Kleinberg et al. 2019. However, here we consider cases of ignorance concerning bias increased or maintained by the technology). And they may not know the precise consequences for those affected by the algorithm. This can be discrimination, for example someone not getting a loan only because he lives in a particular neighborhood, but there are also many other possible effects, some of which are more indirect and “remote”. For example, the developer of an algorithm for financial transactions may not know the consequences for people who are affected by, say, rising food prices as a result of the transactions. And someone may develop an algorithm for image recognition that is first used in an academic context or that is supposed to be used in the medical sector, but is then used for surveillance purposes. And even the precise effects of surveillance technology are not always known to those who develop or employ it, for instance when the technology creates a culture of fear. In that sense, technology is always more-than-instrumental and is used in a social and relational context. But usually neither developers nor users of technology are aware of this.

This raises the question to what extent developers should be aware of the potential alternative uses and misuses of their creations. One could claim that there is a responsibility on the part of the developers to have this awareness. But given the limitations indicated, just claiming that they have this responsibility is not enough. The further question and more interesting question is: how can developers and users be enabled and supported to gain this knowledge? How can this be done in education and in organizations, for example? How can these limits be overcome?

Moreover, developers and users of AI may also be ignorant about their *instrument*. They may know how the technology works, in general, and in this sense know what *means* they use to reach their goal, the Aristotelian end. But this does not mean that they know everything about the technology and the entire chain of actions and things related to it. Some have more knowledge about their instrument than others, and for instance engineers or managers do not necessarily understand everything that statisticians (data scientists) are doing or have been doing before they apply the technology (and vice versa).

This example is also another reminder that AI often involves many people. Unfortunately, neither Aristotle nor many contemporary analytic philosophers included a “with whom” in the responsibility conditions. Like all technological action, action with AI is often collective or, as said, involves many hands along the (time) line. And this is relevant for responsibility. Users of AI and indeed managers or regulators may not always know who else is or has been involved in the development and use of the technology, and what they have been doing and intending. This renders responsible use and responsibility attribution and distribution difficult.

Furthermore, while it is generally recognized that transparency is ethically important for AI and indeed for the use of algorithms and data in general (Mittelstadt et al. 2016), most commentators on AI agree that there is a particular problem with so-called “black-box” AI systems based on machine learning and neural networks. Whereas in the case of classic, symbolic AI the way the technology arrives at a decision is clear by means of, say, a decision tree that has been programmed into the software by domain experts, in the case of these machine learning applications it is *not* clear how exactly the AI system arrives at its decision or recommendation. It is a statistical process and those who create it know in general how it works, but even the developers—let alone further users and those affected by the algorithm, see below—do not know how the system arrives at a particular decision relevant to a particular person. They cannot explain or make transparent the decision making in all its steps.

This lack of transparency and explainability is morally problematic since, to use Aristotelian language again, it creates ‘ignorance’ on the part of the human agents who use the AI. In a sense, the users of these non-transparent systems do not know what they are doing when they use this kind of AI to decide and act for them. In particular, they suffer from the ignorance of *not sufficiently knowing their instrument* (one of the Aristotle’s types of ignorance just mentioned) and therefore from the ignorance of *not knowing what they do* when they give a recommendation to someone based on this kind of AI or when they take decisions or actions based on this kind of AI. For example, when a “driver” of a self-driving car uses the car all the time but does not know how it makes decisions or when a judge uses an AI system for estimating the risk of re-offending of a person and bases her decision about that person on this information, but does not know how it arrived at its advice, there is a sense in which these people do not know what they are doing. This can be seen as constituting at least a morally problematic overreliance on AI, if not a morally blameworthy attempt to offload responsibility. But again, blaming people is not sufficient to address this issue; measures should be taken to avoid this kind of ignorance on the part of the users.

But as said even software developers may not know how exactly the mentioned “black box” systems arrive at their recommendations. Thus, ignorance concerns not only the people affected by AI who might not even know *that* AI is doing things (in the background of an app for example or when they ask a bank for credit) let alone *how* AI works. In the case of non-transparent AI systems, ignorance also pertains to *the expert users*. Note that even in the case of software *in general* there may be developers who do not fully understand the code they are working on (thinking again about the time dimension: most of it may have been written long ago by people who cannot be traced). And they may also not understand the potential *future* uses of their technology or the different domain of application in which their code will be used: Aristotle’s type of ignorance that concerns ‘what or whom he is acting on’. Consider again the example of medical technology that can also be used for military purposes (so-called “dual use”). Even if the developers and people who use the algorithms understand the workings of the algorithm, they might suffer from this blindness for the more remote consequences of their actions.

Thus, with machine learning and other advanced AI specifically, ignorance also hits those who create and use AI technologies, for instance when these technologies arrive at their recommendations or decisions in an insufficiently transparent way. This may be relatively harmless when an AI plays a board game; in other situations, it may be very problematic, even life-changing or lethal. Think of the judge who uses machine learning but is unable to explain why the AI recommends prolonging prison time for a particular person, or the banker who cannot explain why the AI recommends refusing credit to a particular person. But also think of the 737 max pilot who does not know why the airplane’s advanced autopilot system keeps pushing the nose down in spite of his efforts to take control of the airplane. And, in the end, consider the airplane company and the airline who cannot very well explain to the family of the deceased what happened. What is said here is generally true for advanced automation systems, machine learning AI or not: non-transparency and the absence of a sufficient degree of explainability creates a huge problem for the responsible use of AI and other automation technologies.

Now the examples assume that in order to act responsibly, it is important to be able to explain decisions *to* someone, to be able to answer someone who rightfully and reasonably asks “Why?” when given a decision or when acted upon. This *relational* aspect of explainability and responsibility brings us to the next section.

## Include Responsibility Patients, or Responsibility as Answerability: Towards a More Relational View

Often the justification of explainability is neglected. Why precisely is explainability important? It is important for at least two reasons. First, it is needed for the agent of responsibility: in order to act responsibly, to be responsible *for* something, the agent needs to know what she is doing. This was our second Aristotelian condition. Explainability enables the agent to exercise responsible agency. However, there is also a second reason why it is important, which has to do with the “patient” in the responsibility relation. If, influenced by accounts that pay attention to the question “to whom” one is responsible (e.g. Duff 2005), one takes a relational approach to responsibility problems, it becomes clear that there is not only an *agent* of responsibility (the one who acts and who is supposed to act responsibly) but also a *patient* who is affected by the action of the agent (Aristotle’s ‘whom they are acting on’ and others who are remotely affected by this action) and who demands that the agent acts responsibly in the sense that she is expected and asked to (be able to) give reasons for her action. There is the question who is responsible *for* something (responsibility attribution) but also the question who is responsible *to whom*. Responsibility is not only about doing something and knowing what you’re doing; it also means *answerability.* It is also a relational and communicative, perhaps even dialogical matter. The responsibility patient is the addressee, the one who is addressed in the responsibility relation. This role often remains out of sight in standard accounts of responsibility for technology—and responsibility more generally.

Explainability, then, is not only a matter of knowledge on the part of the agent as such (as an Aristotelian condition of responsibility), but can be further justified by saying that the responsibility patient demands an explanation from the responsible agent: the agent needs to be able to explain to the patient why she does or did a particular action, takes or took a decision, recommends or recommended something, etc. For example, one may rightly ask a judge to explain her decision or ask a criminal to explain her actions. Human decisions and actions need to be explainable if they are to be responsible—backward looking and in the present.

Note that the category of responsibility patients does not necessarily exclude non-humans such as animals. Some would even add machines (there is a discussion about whether or not machines can be moral patients, see for example Gunkel 2018b and Bryson 2016). One may well be responsible *to* all kinds of entities if, and to the extent that, they are actually or potentially affected by our decisions and actions. Note also that an ethics that puts the moral patient in a central position is not necessarily focused on, or restricted to, explainability. Consider for example Levinas’s ethics (1969), in which an ethical demand is made that seems to go beyond words, emanating from the “face” of the other. However, to the extent that responsibility is about a more explicit kind of answerability and in so far as we are concerned with *explainability*, the relevant relation is one between humans, who can ask, give, and understand explanations. (And it is also possible that humans demand an explanation *on behalf of* non-humans or indeed on behalf of other humans).

Hence, if a human agent using AI takes a decision based on a recommendation by the AI and is not able to explain why she takes or took that decision, this is a responsibility problem for two reasons. First, the human agent fails to act as a responsible agent *for* what she does because she doesn’t know what she is doing. Second, the human agent also fails to act responsibly *toward* the responsibility patient(s) affected by the action or the decision, who can rightfully demand an explanation for that action or decision since they are affected by it (or, in case the moral patient is not equipped or in a position to make this demand, have others demand that explanation on their behalf).

Ethics of AI, then, should foster the development of AI that supports responsibility on the part of the agents of AI (users, developers) in both of these senses: they should be able to take responsibility for what they do with the AI and should be responsible in the sense of answerable to those affected (or their representatives). With regard to responsibility as answerability, then, what is important ethically speaking is not explainability as a feature of technical systems such as AI; the primary aim is explainability as answerability on the part of the human using and developing the AI. The technical “explainability”, i.e. what the AI system can “say” or “answer”, should be seen as something *in the service of* the more general ethical requirement of explainability and answerability on the part of the human agent who needs a sufficiently transparent system as a basis for the (potential) answers she gives to those affected by the technology. With regard to responsibility for AI, the agent-patient relation is primary, ethically speaking—it is the end; one should then discuss how to shape and support that responsibility relation by technical and other means.

This Aristotelian talk about means and ends *sounds* “instrumental” but does not exclude considering the non-intended consequences of the technology; instead, it requires it. The (explicit) function of technology is to do what it is supposed to do, for example the aim of the autopilot is flying an airplane. But beyond that function, beyond that aim intended by the designers and users of the technology, there is an ethical question about how that technological system impacts the way agents can exercise responsibility *for* this automated flying and *to* those they are answerable to. In the case of the airplane, this is the passengers, in the first place, but also the families, friends and loved ones of the passengers, the other personnel on the airplane, the airline managers, and so on. A relational approach that sees responsibility as answerability opens up an ecology of responsibility relations, which are relevant for responsibility for AI.

Responsible innovation, then, means, among other things, to support the building of AI systems that contribute to the exercise of this kind of relational responsibility. Technical measures can contribute to making AI more explainable. There are researchers who develop techniques for opening the black box (Samek et al. 2017; see also Adadi and Berrada 2018 for an overview of explainable AI methods). Moreover, legal measures could be taken to give people a right of explanation, rather than merely a right of information (e.g. that AI is used and what the AI does), as is now the case in European data protection regulation (GDPR). And responsible innovation also means *asking* people who might be affected by the technology what kind of explanations they need, alongside imagining the ways in which they might be affected (they may be relatively ignorant about the technology and its unforeseeable consequences—a problem that also needs to be tackled).

Listening to people who may be affected by the technology is especially important since, scientifically and philosophically speaking, it is not at all clear what a good explanation consists of, and how much explanation is needed. On the one hand, this renders the goal of explainable AI easier to reach than expected: one should probably not ask for *more* from users and developers of AI than one asks from *anyone else*. Research on explanation has shown that generally people do not provide or expect complete causal chains, select explanations, and respond to what they believe are the beliefs of the other. Explanations are social (Miller 2019). On the other hand, this social and relational understanding of explanation makes the goal of responsible AI understood as explainable AI *harder* to reach, namely if the aim is to have AI explain things to humans: AI may not sufficiently “understand” the social.

Yet if the goal is not to have *machines* explain, but rather demand this from *human beings* who are able to explain things to other human beings, then there is a chance that explainable AI may work. Responsibility then does not mean that one explains *everything* that contributed to the action or decision, but rather that one can know and select what is relevant to what the other (human being) wants and needs to know. In the case of AI and other advanced automation systems, this *can* be done by humans if and only if (a) those humans are sufficiently supported by technical systems that are transparent *enough* for the (primary) purpose of humans explaining things to other humans (there is no demand for absolute transparency here), and (b) those humans are sufficiently willing, capable, and educated to imagine and understand what those affected by the technology may ask and demand from them. This can be supported by actually asking stakeholders what kind of explanations they actually want and need. The assumption is then that only humans can really explain and *should* explain what they decide and do, and that explanation itself is deeply social and relational.

But do people need explanations or do they need reasons? Can explanations count as reasons, and, if so, when? Responsibility as answerability can also be formulated in terms of reasons, or more specifically, in terms of *giving* reasons. And then for reasons the same seems to hold as for explanations in general: assuming that only humans can really give reasons, then responsible AI means that humans should get this task. The development of AI should then support this human task of giving reasons to those who ask or may ask questions about the actions and decisions mediated by the technology. If we, as a society, want to respect human beings as not only autonomous but also social human beings, developers and users of AI owe those affected by AI an answer: the latter rightly demand reasons and explanations for actions and decisions that affect them. Responsible agency with regard to AI for developers and users, then, means not only to control things and to know things in a particular way, but also to answer, explain, give reasons, and communicate. These aspects of responsible AI and responsible technology are not just side issues but ethical requirements. Technology development and technology education should be altered in ways that better support users and developers of AI in answering the “Why?” question. Moreover, society should reflect on whether to allow a high degree of automation in cases where the high speed and volume of decisions, for example in military cases or in the case of high frequency trading, make it impossible to answer or reason, since the “Why?” always comes too late.

Finally, a more relational understanding of responsibility can also help to ask further questions that address the many hands problem and are critical of the emphasis on individual control in traditional theories. For example, maybe some problems can be solved by pointing not only to distributed responsibility but also to *collective* responsibility. Collective responsibility can mean two things: that the responsibility is distributed over a set of individual actors (in the sense that there is a set of individual agents who each bear responsibility), or that a *collective agent* such as a community or an organization is held responsible. But while talking about “collective agency” might make sense, it is far more controversial to talk about “collective responsibility.” This deserves its own discussion. One could also explore and discuss whether there are cases in which a society as a whole (or all citizens, or even an entire culture) constitutes the agent of responsibility, as well as the patient. For example, if there is a problem with bias in an AI algorithm, but this bias is present in the language (e.g. English), then it seems that there is responsibility not only on the part of the current language users as individuals but also the entire community of language users and the culture, both in the present and in the past. Again there is a time dimension: the historical use and development of a particular language—as connected with various histories and people—has effects on current language users and, indirectly or directly, on the actions of the AI and its consequences. It seems very difficult to deal with this if one only considers individual responsibility. For example, there may be bias that is present in language corpora: if a machine learning AI is trained on texts from the web, it may reproduce this historic bias, e.g. gender bias (Caliskan et al. 2017). To do something about this kind of bias, it seems that individual action and conceptual frameworks of individual responsibility are insufficient, even if it is not clear what collective responsibility means.

It should also be noted that in the light of this social, cultural, and historical context, responsibility probably cannot and should not always be *absolute*: even if there are voluntary actions of humans using technologies such as AI, there are many circumstances, histories, institutions, etc. which go beyond our individual or even collective *full* control, but which nevertheless shape what technologies do and become. In so far as this is the case, responsibility for AI and other technologies may be limited to some degree and has a tragic aspect (Coeckelbergh 2011). This tragic dimension includes tragic choice: in contrast to what utilitarian or deontological discussions of Trolley dilemmas assume, AI technology may create dilemmas that cannot be resolved. For instance, in the case of self-driving cars there may be deadlock situations; one should recognize the tragic dimension of this (Sommaggio and Marchiori 2018). However, this does *not* mean that in such cases responsibility cannot or should not be ascribed at all, and that nothing can or should be done to deal with the problems. For example, if there is bias in terms of race or gender in a dataset or algorithm, this has a collective aspect since it might be partly in our language and a tragic aspect since humanity and society may never be able to eradicate unjust bias, but in the context of a particular use of AI, it could be at least partly corrected for by the technical system or by the humans using the AI. And if the use of self-driving cars with AI happens to lead to unresolvable dilemmas, then responsible development could mean that this problem, its tragic dimension, and how to deal with it should be discussed upfront and publicly, with stakeholders and in society at large, rather than giving the impression that the technology or indeed philosophical normative theory can provide the “correct” answer. A relational approach to responsibility should never be an excuse for evading responsibility but instead should highlight the need for responsible action understood as having the aspects of *interaction*, dialogue, transparency, and understanding—including understanding the tragic dimensions of technological action.

## Conclusion: Responsibility for AI

This was an overview of some problems concerning responsibility for AI, with a focus on the problem of responsibility attribution and responsibility as answerability. We moved from a standard discussion about control and knowledge by moral agents who use and develop AI to a more relational view, which considers not only the moral *agents*’ degree of control and awareness of unintended consequences and moral significance of their actions, for instance, but also the moral patients affected by AI: those who may demand and deserve an answer concerning what is done to them and decided about them with and through AI. In the course of my arguments, I have stressed the temporal dimension: the causal chains and the operations, but also the history of the societies in which the technology development and use are embedded. Since AI systems are already used today, these concerns are not only philosophically interesting but are also very practical and urgent. In so far as AI is already pervading our daily lives, *all* people are the moral patients talked about in this discussion. If the conceptual framework offered here makes sense, then moral patients are justified in demanding AI technology and social arrangements that enable effective attribution and distribution of responsibility and requiring relevant agents to exercise their responsibility when using and developing AI. Using the relational framework presented here, this requirement to exercise responsibility means that society deserves AI experts and operators who are in control, know what they are doing, and are *able and willing to communicate, explain and give reasons for what they are doing* to human and nonhuman moral patients. This includes the obligation to gain greater awareness of unintended consequences and the moral significance of what they do, including how they deal with tragic problems. If AI is not going to be responsible in this sense, it will crash.

## Footnotes

1 https://www.theguardian.com/world/2019/mar/20/lion-air-pilots-were-looking-at-handbook-when-plane-crashed.

2 https://www.extremetech.com/extreme/268915-fatal-arizona-crash-ubercar-saw-woman-called-it-a-false-positive.

3 http://synergiainsights.in/morning-brief/lion-air-610-crash-faulty-sensors-not-fixed.
